# Advanced Midwifery Practice in Switzerland: Development and challenges

**DOI:** 10.18332/ejm/185648

**Published:** 2024-04-22

**Authors:** Eva Cignacco, Anja Schlenker, Silvia Ammann-Fiechter, Therese Damke, Claire C. de Labrusse, Astrid Krahl, Barbara Stocker Kalberer, Andrea Weber-Käser

**Affiliations:** 1 School of Health Professions, Bern University of Applied Sciences, Bern, Switzerland; 2 Institute of Midwifery and Reproductive Health, ZHAW Zurich University of Applied Sciences, Winterthur, Switzerland; 3 HESAV School of Health Sciences, HES-SO University of Applied Sciences and Arts Western Switzerland, Lausanne, Switzerland; 4 Swiss Federation of Midwives, Olten, Switzerland

**Keywords:** midwifery, advanced practice, advanced midwifery practice, perinatal healthcare

## Abstract

Midwifery is undergoing increasing complexity attributed to global epidemiological, socio-economic and technological shifts. Coupled with a shortage of workforce and the imperative for cost-effectiveness and high-quality care, there is an ongoing international discourse and establishment of new care models and specialized roles, notably Advanced Midwifery Practice (AMP). While countries like the UK and Ireland have embraced AMP roles, Switzerland lags behind with only a few pioneering roles. The absence of regulatory frameworks for AMP within the Swiss legal and healthcare system, hinders the evolution of APM roles necessary to address contemporary needs in perinatal healthcare provision. To effectively harness the midwifery workforce and mitigate premature attrition, Switzerland must formulate distinct career trajectories for postgraduate midwives, particularly for Advanced Practice Midwives (APM). This involves establishing legal standards for educational and clinical prerequisites, delineating guidelines for APM responsibilities and competencies, and devising compensation schemes that mirror the autonomy and leadership competencies integral to these advanced roles within inpatient and outpatient perinatal care models. The incorporation of evaluation and research into AMP is indispensable, contributing to improved patient outcomes and the ongoing professionalization of midwifery. In conjunction with the Swiss Federation of Midwives, all Universities of Applied Sciences in Switzerland have collaboratively drafted a national position paper underscoring the significance of developing APM roles to ensure the provision of high-quality perinatal care. This article aims to elucidate current developments in perinatal care within the Swiss context, providing a comprehensive definition for AMP, delineating its contribution to enhancing and sustaining the quality of care.

## INTRODUCTION

Midwives constitute indispensable healthcare providers for ensuring the provision of high-quality maternal and neonatal care^1,2^, not only in low- and middle-income but also in high-income countries such as Switzerland^3^. Their pivotal role extends to the promotion of sexual and reproductive health education and care, thereby contributing to improved outcomes^3,4^, and facilitating the achievement of Sustainable Development Goals^3^. In the face of increasing complexity in perinatal care, midwives are now more than ever called upon to adhere to evidence-based practices and assume leadership roles within autonomous midwifery care models or interdisciplinary teams. In the European context, the UK and Ireland have been at the forefront of establishing standards and requirements for APM, thereby regulating their competencies and delineating job descriptions. While Switzerland has seen the emergence of initial APM roles requiring a Master’s degree, the absence of formal regulation remains a notable gap.

This article aims to elucidate current developments in perinatal care within the Swiss context, providing a comprehensive definition for Advanced Midwifery Practice (AMP) and delineating its contribution to enhancing and sustaining the quality of care. Additionally, it delves into the prevailing challenges associated with the establishment of new APM roles and proposes strategic actions to foster high-quality and cost-efficient perinatal care.

## COMMENTARY

### Developments in midwifery care

Over the preceding decades, the field of midwifery has undergone a notable increase in complexity. Many epidemiological, societal, and technical developments are influencing midwifery as a profession. These trends also impact the practice of midwifery within the field of perinatal care. In this article, perinatal care refers to the comprehensive support provided throughout pregnancy, childbirth, the postpartum period, and the first year of the child’s life, focusing on the well-being of the mother, newborn, and family^3^,^5-8^.

Primarily, epidemiological trends manifest a notable increase in chronic diseases and multimorbidity across all age groups. This includes a surge in conditions such as obesity, cardiovascular, endocrinological, and hematological diseases^3^,^6^. Moreover, mental health disorders during the perinatal period necessitate midwives to possess augmented and specialized knowledge^9-11^ to effectively screen, identify, counsel, and appropriately refer affected women to specialized care. The recent SARS-CoV-2 pandemic has contributed to a range of adverse pregnancy outcomes, encompassing pre-eclampsia, preterm birth, and stillbirth^12^. Additionally, the pandemic has resulted in a sustained reduction in the midwifery workforce^3^. These trends collectively amplify the likelihood of adverse outcomes, underscoring the imperative for adapted models of care during pregnancy and childbirth^13^.

Secondly, socio-economic determinants are marked by an increased prevalence of women with a migrant background^14^,^15^, delayed maternal age at childbirth^16^, and residence in socially deprived conditions^17,18^. These phenomena are correlated with elevated rates of adverse maternal and neonatal outcomes. The promotion of equal opportunities for families in these vulnerable circumstances necessitates additional competencies for midwives in diversity and ethical decision-making^19^.

Thirdly, advancements in technology, particularly in genetics and reproductive, fetal, and neonatal medicine, demand specialized knowledge and competencies in counseling and in the principles of shared ethical decision-making for midwives^19^.

Fourthly, evolving expectations and demands from women and their families signal a shift toward participatory and woman-centered care, with a focus on preserving and promoting physiological processes during pregnancy, childbirth, and the postpartum phase. Additionally, health promotion and preventive measures undertaken by midwives during early childhood warrant emphasis^3^. Robust evidence indicates that midwifery-led models offer continuous and safe care, resulting in high maternal satisfaction and fewer adverse outcomes^1,20,21^. Pre- and post-pregnancy interventions that place in the foreground the woman herself, rather than solely the pregnancy, play a significant role in reducing the risk of adverse maternal outcomes, highlighting the necessity for well-skilled multidisciplinary teams throughout the continuum of care of pre-pregnancy, pregnancy, and post-pregnancy^5^. The cost-effectiveness of midwifery-led care has been demonstrated for low-risk pregnancies^22-24^, underscoring the need for further systematic research^25^.

Finally, there is a projected shortage of 1.1 million qualified health workers, primarily midwives, to meet global health needs by 2030^3^. A study on Swiss hospitals predicts a shortage of 30500 nurses in 2030, attributed to various factors^26^. More precise data regarding midwives in Switzerland are lacking due to the relatively small size of this professional group^27^. Among these factors, insufficient education and training, including supervision and mentoring, and inadequate regulation, identified by the UNFPA^3^, contribute to poor-quality care in the perinatal period. The COVID-19 pandemic has exacerbated staffing shortages, with lasting effects expected due to factors such as redeployment^3^. Ensuring continuity of care and a safe environment for families amidst increasing complexity, necessitates enhanced communication skills, explanatory capabilities, and effective interprofessional collaboration^3,7^.

### Advanced Practice Midwifery in Europe: Definition and core capabilities

In Ireland and the UK, the Advanced Practice (AP) model, as described by Hamric and Hanson^28^, is a well-established educational concept in the nursing profession^29^. Unlike the nursing profession, midwifery is characterized by a high degree of autonomy, exhibits diverse titles, roles, and practice activities for APM internationally^30^. Existing literature lacks an international standard for advanced midwifery scope of practice^31^.

Some APM roles focus on extended practice, while others integrate leadership, teaching, and research. Importantly, to the best of our knowledge, there is no universally valid definition of Advanced Practice Midwifery^20^. In the year 2016, Goemaes et al.^20^ defined the ‘revolutionary concept’ of APM as ‘a level of midwifery practice at which midwives use their expertise, management, and clinical leadership skills to provide evidence-based, tailored care for women and their families independently and autonomously. Professional leadership and research skills are used to evaluate practice and advance midwifery as a profession and science’.

Aligned with the Swiss Federal Office of Public Health’s promotion of interprofessional approaches in healthcare and the distinction between Bachelor’s (BSc) and Master’s (MSc) degrees^32^, the Swiss Midwifery Education Board, in collaboration with the Swiss Federation of Midwives^33^, formulated a supplementary definition: ‘An Advanced Practice Midwife is an accredited practicing Midwife with an MSc degree, in-depth expertise in a specific practice domain, research skills, and advanced leadership competences. Advanced Practice Midwives provide continuous woman and family-centered care in complex clinical situations with a high degree of autonomy, efficacy, and accountability. They work in various settings, promoting and coordinating interprofessional collaboration within the health and social system. Advanced Practice Midwives contribute to the production of scientific knowledge, communicate it to diverse audiences, and conceptualize and implement accessible, equitable, cost-effective, and innovative solutions for health promotion and prevention. They improve the quality of care, contribute to public health, and advance midwifery as an academic profession’.

In light of limited international consensus on the definition, there exists a paucity of literature on Advanced Practice Midwifery, primarily due to the scarcity of midwifery roles explicitly designated as advanced practice within their titles or role descriptions^8^. Challenges have also arisen in accurately gauging the impact of APM roles^24^. Healthcare policy-makers posit that specialists and advanced practitioners contribute to elevated care quality^34^, particularly at a strategic level^35^. Although the positive impact on clinical, strategic, and financial outcomes can be presumed, further research on the effects of APM roles is imperative^20,25^. Within the perinatal setting, Brooten et al.^36^ demonstrated that home-based care for high-risk pregnancies, provided by specialized midwives, resulted in fewer preterm births and reduced hospitalization days, leading to cost savings. Sandall et al.^37^, in their systematic review, noted a lower incidence of Intrauterine Fetal Death (IUFT) in association with APM roles^37^. Casey et al.^25^ highlighted in their review that the utilization of APM improved access to primary care in obstetrics, enhanced outcomes for selected client populations, and resulted in reduced costs.

Ireland has been at the forefront of developing specialized roles for midwives, particularly APM, which require specific training and practical experience^34^. In 2018, the Nursing and Midwifery Board of Ireland (NMBI) delineated competencies, standards, and requirements essential for APM roles^38^, thereby facilitating their establishment^39^. The competencies encompass a broad spectrum, including advanced assessment and intervention strategies, making research-based clinical decisions, analyzing complex interactions, guiding decision-making, and developing client-focused care. The implementation of APM roles, as recognized by the NBMI^38^, necessitates attention to leadership, infrastructure, and education.

In November 2022, the National Health Service (NHS) of the UK followed suit by releasing the ‘Advanced Clinical Practice in Midwifery: Capability Framework’^40^. This framework outlines three prerequisites for individuals aspiring to become APM, namely:

1. Registration as a midwife on the NMC Register.
2. The four generic capabilities for Advanced Clinical Practice, as defined in 2017 in the ‘Multi-professional framework for Advanced Clinical Practice in England’ by Health Education England (HEE)^41^, encompassing Clinical Practice, Leadership and Management, Education and Research.
3. APM-specific core capabilities^40^, such as demonstrating clinical autonomy, authority to complete episodes of complex maternity care (including prescribing), possessing the agency, knowledge, and skillset to overcome organizational and cultural barriers, providing collegiate approaches to transparent care planning, acting as a role model and facilitating reciprocal multiprofessional and multidisciplinary team learning, identifying organizational needs, utilizing quality improvement methodology, and actively participating in research. These skills and knowledge are to be acquired through formal education and training in the clinical environment, supported by a structured program and mentorship^40^.

The APM Capability Framework acknowledges midwives as the lead professionals for the care and support of women and newborn infants, emphasizing the opportunity for midwives to develop their skills and knowledge in various directions. Development, starting from the core capabilities, can occur across any or all of the four domains of advanced practice^40^.

### Advanced Practice Midwifery in Switzerland

In Switzerland, no official regulation or definition of competencies and prerequisites for APM roles currently exists. Regulation for healthcare professions, such as nurses, physiotherapists, or midwives, is established at the BSc level within the legal framework of the Federal Act on Health Professions^42^. The absence of MSc-level regulation poses a significant barrier to establishing a national professional register for APM, implementing AP roles, and determining compensation^33^.

The Federal Office of Public Health (FOPH) is addressing workforce challenges, particularly for nurses, within the national care initiative (‘Pflegeinitiative’). The FOPH acknowledges that Advanced Practice Nurses (APN) possess higher academic education, expert knowledge, decision-making skills for complex situations, and clinical competencies for advanced nursing practice. Discussions on state-regulated degrees or continuing education for APN are underway within the second stage of the ‘Pflegeinitiative’ until early 2024^43^.

While APN in Switzerland work in highly specialized areas, regulation and standardization for APM roles lag significantly behind those in the UK and Ireland. Nonetheless, initial APM models have been established^9,44^.

### Case Vignette: APM Perinatal Mental Health at Clinic for Obstetrics and Gynaecology, Inselspital, Bern

The APM for Perinatal Mental Health at the Clinic for Obstetrics and Gynaecology, Inselspital, Bern, collaborates closely with the University Psychiatric Services Bern. The investigation of Sutter^9^, inspired by care models in Ireland and England, addressed the need for an adapted model of care in perinatal mental health at the Clinic for Obstetrics and Gynaecology. Employing a participatory, evidence-based, woman-focused process, the demand for the adapted model of care was identified through interviews with affected women and experts. Results were presented to the Departments of Obstetrics and Psychiatry, leading to optimized care, the establishment of the APM role, and new services.

The APM tasks include strengthening perinatal mental health, promoting early mental health, early detection of symptoms of mental disorders, counseling affected women and families, and supporting professional colleagues and other non-medical health professionals. While the APM do not make psychiatric diagnoses or offer psychotherapy, coordinating treatment plans is a crucial responsibility, given the involvement of various specialists in the perinatal period. Sutter (Email, September 6, 2023) emphasizes that for an AP role, in-depth expertise in the specific field, coupled with solid project management, clinical leadership, and research skills, is crucial.

### Discussion of challenges for APM implementation in Switzerland

Few countries have explicitly outlined standards and guidelines for Advanced Practice Midwifery, a situation evident in Ireland and increasingly also in the UK in recent years. Frequently, there is a notable ‘inconsistency regarding policy, education, titles, roles, scope of practice, skills, and competencies’, as described by Egerod et al.^45^, concerning advanced roles in critical care nursing in Europe. The UNFPA^3^ reports that only half of reporting countries have midwife leaders within their national Ministries of Health. Regrettably, to date, there has been no appointment of a chief midwife officer within either federal or cantonal policy entities. Limited opportunities for midwives to assume leadership positions and the scarcity of female role models in such positions, impede midwives’ career progression, advancement, and their ability to realize their full potential.

In the Swiss context, the following five deficiencies in regulation and strategy are deemed crucial.

*Lack of a legal basis and regulation*

As indicated earlier, the Federal Assembly of the Swiss Confederation, in 2016, opted not to legally regulate the Master of Science (MSc) level for most health professions^7,42^. Resistance from politicians and health insurers to further academicize non-medical health care professions and expand their professional roles was a primary reason. The absence of a legal framework at the MSc level for health professions poses a significant obstacle to establishing a national professional register for APM, implementing AP roles in general, and determining their remuneration^33^.

*Need for a clear APM definition*

A clear definition of APM, as provided by the Swiss Midwifery Education Board and the Swiss Federation of Midwives in its national position paper^33^, is crucial for regulation within the framework of the Health Insurance Act^46^. This definition is essential for fostering discourse within the midwifery profession and promoting its ongoing professionalization. Building upon this, a national discourse involving collaboration among academic midwives should enhance understanding of the contribution and necessity of APM role development for sustaining high-quality care in the perinatal period.

*Lack of defined requirements for the title APM*

In Switzerland, numerous graduates of continuing education programs with Certificates (CAS), Diplomas (DAS), or Master of Advanced Studies (MAS) often partially assume AP roles in practice. To address this, the expert group of the nursing profession has proposed a transitional solution of 10 years, allowing health professionals with CAS, DAS, or MAS degrees the opportunity to fulfill formal training requirements or pursue bridging programs to obtain the MSc degree. The absence of legal requirements contributes to role uncertainties, as demonstrated by Eissler and Zumstein-Shaha^47^ in their analysis of Advanced Practice Nurses (APN) in Switzerland. The Swiss Midwifery Education Board and Swiss Federation of Midwives^33^ currently suggest the following regulations: candidates for APM should hold an MSc degree in Midwifery or a related field of health and have a minimum of 5500 hours of professional experience as a practicing midwife before or until obtaining the MSc degree. This professional experience may be part-time but must be equivalent in total to the specified number of hours. Moreover, to be registered as APM or retain the title, the candidate must work at least 40 to 50 percent as APM in clinical practice, with the majority of the workload involving direct patient contact.

*Lack of differentiation of competences between BSc and MSc midwives and APM*

In Switzerland, BSc programs in Midwifery were established in 2008. Since 2017, MSc programs in Midwifery have also been offered in the country^33^. As of 2022, 192 midwifes held a higher academic degree than a BSc, specifically an MSc or higher. This constitutes approximately 5 percent of the total of roughly 3840 registered midwives in Switzerland^27^. These MSc programs teach core competences of Advanced Practice (AP) as defined by Tracy et al.^28^, including counseling, evidence-based practice, leadership, collaboration, ethical decision-making, and team coaching. However, confusion persists in practice regarding the clear distinction between BSc and MSc competencies. There is a significant need for discussion about the added value of midwives with an MSc in an AP role concerning perinatal healthcare for women, children, and their families. In November 2023, after more than a year of collaborative work, the Swiss Midwifery Education Board approved the common set of MSc competences for health professions, including midwives (unpublished). This set of competences is based on the Canadian CanMeds network, analogous to the BSc core competences of midwives. Additionally, midwifery-led research conducted at Swiss Universities of Applied Sciences contributes to improving midwifery care and, consequently, the health outcomes of mothers and infants.

*Lack of national cost data and remuneration systems*

Reliable cost data on Advanced Midwifery Practice (AMP) roles in Switzerland, both in the inpatient and outpatient sectors, are unavailable. Although Advanced Nurse Practitioner (ANP) role profiles in the inpatient setting are widespread, there is a lack of public cost data. In evaluating four outpatient settings, Gysin et al.^48^ found a lack of remuneration systems. In the PriMA project in the Swiss canton of Bern, Schlunegger et al.^49^ evaluated the function and costs of Advanced Practice Nurses (APN) in primary care. They emphasize the need for recognizing the APN service as independent and billable to support primary healthcare sustainably^49^. In the Swiss canton of Basel, Schwind et al.^18^ evaluated the impact of *de facto* APM care within the SORGSAM (Support at the Start of Life) project in 2023 for families in vulnerable situations. The authors demonstrated how offering improved assistance to psychosocially and economically disadvantaged families enhances parental resources, leading to better postpartum health outcomes for mothers and children. Despite being funded by a foundation, the services of this piloting project were not reflected in healthcare compensation schemes. Defining specialist services by APM in inpatient and outpatient settings should result in billability that reflects autonomy and responsibility levels, enabling further research and evaluation of the impact of AMP on cost-effectiveness.

### Recommendations for sustaining high-quality midwifery care with APM roles in Switzerland

To uphold high-quality perinatal care amid increasing complexity and workforce shortages, the development of Advanced Practice Roles in healthcare is imperative^3,40^. APM play a crucial role in creating sustainable perinatal care that responds to evolving needs. Policymakers and educational institutions must address epidemiological and socioeconomic challenges to ensure the availability of highly qualified and specialized midwives. Proposed actions include political regulation, organizational planning, and ongoing evaluation of APM models of care and research^40^.

## CONCLUSION

In the face of heightened complexity, workforce shortages, and the imperative for cost-effective healthcare, new models of perinatal care are essential. APM are pivotal in developing, implementing, and evaluating these models. National health authorities and educational institutions must tackle epidemiological and socioeconomic challenges to ensure the availability of highly qualified midwives. Piloting projects demonstrate that APM can be a cost-effective model in Swiss perinatal care, contributing not only to the healthcare system but also to the advancement and academization of health professions. APM roles, with their varied focuses, provide opportunities for midwives to develop in different directions, enhancing the attractiveness of the health professions. Collaborative efforts by policymakers, maternity organization leaders, and researchers are essential to overcoming obstacles hindering the development of APM models of care.

## Data Availability

Data sharing is not applicable to this article as no new data were created.
